# Tubulin binding cofactor C (TBCC) suppresses tumor growth and enhances chemosensitivity in human breast cancer cells

**DOI:** 10.1186/1471-2407-10-135

**Published:** 2010-04-12

**Authors:** Rouba Hage-Sleiman, Stéphanie Herveau, Eva-Laure Matera, Jean-Fabien Laurier, Charles Dumontet

**Affiliations:** 1 Inserm U590, Laboratoire de Cytologie Analytique, Université Lyon 1, 69008 Lyon, France

## Abstract

**Background:**

Microtubules are considered major therapeutic targets in patients with breast cancer. In spite of their essential role in biological functions including cell motility, cell division and intracellular transport, microtubules have not yet been considered as critical actors influencing tumor cell aggressivity. To evaluate the impact of microtubule mass and dynamics on the phenotype and sensitivity of breast cancer cells, we have targeted tubulin binding cofactor C (TBCC), a crucial protein for the proper folding of α and β tubulins into polymerization-competent tubulin heterodimers.

**Methods:**

We developed variants of human breast cancer cells with increased content of TBCC. Analysis of proliferation, cell cycle distribution and mitotic durations were assayed to investigate the influence of TBCC on the cell phenotype. *In vivo *growth of tumors was monitored in mice xenografted with breast cancer cells. The microtubule dynamics and the different fractions of tubulins were studied by time-lapse microscopy and lysate fractionation, respectively. *In vitro *sensitivity to antimicrotubule agents was studied by flow cytometry. *In vivo *chemosensitivity was assayed by treatment of mice implanted with tumor cells.

**Results:**

TBCC overexpression influenced tubulin fraction distribution, with higher content of nonpolymerizable tubulins and lower content of polymerizable dimers and microtubules. Microtubule dynamicity was reduced in cells overexpressing TBCC. Cell cycle distribution was altered in cells containing larger amounts of TBCC with higher percentage of cells in G2-M phase and lower percentage in S-phase, along with slower passage into mitosis. While increased content of TBCC had little effect on cell proliferation *in vitro*, we observed a significant delay in tumor growth with respect to controls when TBCC overexpressing cells were implanted as xenografts *in vivo*. TBCC overexpressing variants displayed enhanced sensitivity to antimicrotubule agents both *in vitro *and in xenografts.

**Conclusion:**

These results underline the essential role of fine tuned regulation of tubulin content in tumor cells and the major impact of dysregulation of tubulin dimer content on tumor cell phenotype and response to chemotherapy. A better understanding of how the microtubule cytoskeleton is dysregulated in cancer cells would greatly contribute to a better understanding of tumor cell biology and characterisation of resistant phenotypes.

## Background

Microtubules are crucial structures for living cells as they are involved in many biological functions including cell motility, cell division, intracellular transport, cellular architecture as well as other cell types specific functions [1]. Their dynamic property involved in cell division makes out of microtubules major targets for anticancer drugs. The drugs commonly used are divided into two main families of taxanes and vinca alkaloids which are known to suppress microtubule dynamics by stabilizing or destabilizing the microtubules respectively and thus inhibiting the metaphase anaphase transition, blocking mitosis and inducing apoptosis [2]. Taxanes and vinca alkaloids are among many drugs used to treat breast cancer [3]. However, resistance to anticancer drugs is appearing, inducing a need to understand and identify the mechanisms behind of it. One of the mechanisms responsible of the resistance phenotype is the alteration in the dynamic properties of microtubules. Microtubules have two main dynamic behaviors. First they exhibit a dynamic instability which consists of apparently random transitions between slow elongation and rapid shortening states [4]. Another important property is the treadmilling by which tubulin subunits continuously flux from one end of the polymer to the other, due to net differences in the critical subunit concentrations at the opposite microtubule ends [5].

Microtubules are made of α/β-tubulin heterodimers whose proper folding involves many chaperonins as well as protein cofactors [6,7]. After being synthesized, the tubulins are sequestered by cytosolic chaperonins for their correct folding and preparation for further interactions with the tubulin binding cofactors TBC [8,9]. The proper folding pathways of α-tubulin and β-tubulin into the dimers are interdependent and five TBC (TBCA to TBCE) are involved. TBCB and TBCA bind to α- and β-tubulins respectively and the formed complexes serve as reservoirs of tubulin peptides in the cytoplasm [10,11]. The α- and β-tubulins are then delivered to TBCE and TBCD respectively where they form a supercomplex with TBCC (TBCE/α-tubulin/TBCC/TBCD/β-tubulin). After hydrolysis of GTP by β-tubulin, this complex releases activated α/β-tubulin heterodimers which can readily polymerize into microtubules [10,12,13].

Little is known regarding the role of TBCs in cancer. It has been shown that the inhibition of TBCA in MCF7 and HeLa cells modified the microtubule structures, caused cell cycle arrest in G1 and cell death [14]. TBCB and TBCE have been found to physically interact and induce microtubule depolymerization *in vitro *[15]. In addition to their roles in the proper folding of microtubules, these cofactors might have other roles involving microtubules. It was recently shown that microtubules contribute to the mechanism of cell detachment through TBCD by transporting it to the cell membrane where it interacts with adherent and tight junctions [16]. It has been described that TBCD interacts with Arl2, ADP ribosylation factor like 2 (Arl2) which dissociates it from the α/β tubulin heterodimers [17]. A study in *Arabidopsis thaliana *showed that TBCC plays an important role in releasing competent α/β-tubulin polymerizable heterodimers [18,19]. Another study performed in X-linked retinitis pigmentosa 2 (RP2) showed that RP2 responsible for the progressive degeneration of the photoreceptor cells and TBCC have similar sequences. Both of these proteins were found to be activators of GTPases but only TBCC is capable of catalyzing the heterodimerization of tubulins [20].

Since TBCC is crucial for the proper folding of tubulins and their polymerization into microtubules and since little is known about this protein with respect to breast cancer, we were interested in studying the impact of TBCC overexpression on the phenotype of tumor cells as well as on microtubule content and dynamics and response to antimicrotubule drugs. We have found that overexpressing TBCC influenced cell cycle distribution of breast cancer cells in our model along with an increase in percentage of cells in G2-M phase of cell cycle and a slower mitosis. The dynamics of microtubules were reduced and the content of polymerizable tubulins was decreased. Finally, cells overexpressing TBCC were more sensitive to microtubules targeting agents both *in vivo *and *in vitro*.

## Methods

### Plasmid construction

The pcDNA6/V5-His A plasmid was used to clone the 1 kb human *TBCC *cDNA (NM_003192) extracted from hTerT-HME-1 human mammary epithelium cells (ATCC). The mRNA was extracted using Trizol reagent (Invitrogen, Cergy Pontoise, France) following the manufacturer's instructions. Reverse transcription into cDNA was then done using Moloney leukaemia virus reverse transcriptase (Invitrogen, Cergy Pontoise, France) for 1 hour at 37°C as described in the manufacturer's manual. The cDNA obtained was then amplified by the full length *TBCC *forward GCCAATATGGAGTCCGTCAG and reverse CAACTGCTTAGTCCCACTGGA primers using a high fidelity polymerase according to the manufacturer's instructions (Jena Bioscience, Germany). The PCR conditions were 30 cycles of denaturation at 94°C for 1 min, annealing at 60°C for 1 min and elongation at 72°C for 1 min. Purified amplicon was subcloned into pGEMTeasy and thereafter into pcDNA6 in the sense orientation (designated as pcDNA6/C+) using EcoRI (Fermentas, France).

### Cell culture and transfections

MCF7 (human mammary adenocarcinoma, ATCC) cells and transfectants were grown in DMEM supplemented with penicillin (200 UI/ml), streptomycin (200 μg/ml) and fetal bovine serum (10%) at 37°C in a humidified atmosphere containing 5% CO_2_. Cells were transfected with pcDNA6/C+ or empty pcDNA6 using lipofectin (Invitrogen, Cergy Pontoise, France) following the manufacturer's instructions. The stable transfectants were obtained through blasticidin selection (20 μg/ml) (KN-1004, Euromedex, France). Cloning of the populations of cells was performed for each of the two batch populations and 3 clones representative of each population were selected for further characterization, on the basis of their differential levels of expression of the protein TBCC. The clones designated MC+1, MC+2 and MC+3 represent the clones overexpressing TBCC. The clones designated MP6.1, MP6.2 and MP6.3 represent the control clones.

### Western blot analysis

Protein extraction and western blot analysis were performed as described previously [21]. The antibodies used were anti β III-tubulin (clone Tuj1, 1/2500; Covalab, Lyon, France), anti p53 (clone DO7, 1/1000; Dako, Denmark), anti β-actin (clone AC-15, 1/5000), anti α-tubulin (clone DM1A, 1/1000), anti β-tubulin (clone 2.1, 1/1000), anti tyrosinated α-tubulin (clone TUB-1A2, 1/1000) and acetylated α-tubulin (clone 6-11B-1, 1/1000) from Sigma Aldrich (St Quentin Fallavier, France). The polyclonal antibodies against TBCC (1/800) and TBCD (1/3000) were generously provided by N. Cowan (New York University Medial Center, USA) and those against Arl2 (1/1000) and Glu-tubulin (1/1000) were generously provided by R. Kahn (Emory University School of Medicine, Atlanta, USA) and L. Lafanechère (Centre de Criblage pour des Molécules Bio-Actives, Grenoble), respectively. Expression levels of the proteins were standardized against the β-actin.

### RNA interference assays

A desalted duplex siRNA targeting *TBCC *5'-CUGAGCAACUGCACGGUCA-3' and its corresponding scrambled sequence were designed by Sigma-Aldrich (St Quentin Fallavier, France). The siRNAs (200 nM) were transfected into 25 × 10^4 ^of MCF7, MP6.1 or MC+1 cells using oligofectamine (Invitrogen, Cergy Pontoise, France) according to the manufacturer's protocol on two consecutive days. Protein analyses by western blot or flow cytometry experiment were done on the third day after transfection.

### Cell proliferation analysis

Cell proliferation was estimated using the methylthiazoletetrazolium (MTT) and BrdU assay. For the MTT test, cells (13 × 10^4^) were seeded in a 6-well plate and incubated at 37°C. Every 24 h and for one week, MTT (3 mg) was added to each well of each plate. After 2 hrs of incubation at 37°C, supernatants were removed, formazan crystals solubilized with 3 ml of isopropanol-HCl-H_2_O (90:1:9, v/v/v) and plates scanned. The absorbance was measured spectrophotometrically with a microplate reader (Labsystem Multiskanner RC) and the MTT values were obtained as subtraction of absorbances read at 540 and 690 nm wavelengths. The nonradioactive BrdU-based cell proliferation assay (Roche, Basel, Switzerland) was performed according to the manufacturer's protocol. Treated and untreated cells (5 × 10^3 ^cells per well) were seeded in a 96-well plastic plate and the assay was performed after 48, 72, 96 and 168 hours. Treated cells were exposed to either 0.5 nM or 1 nM of gemcitabine (Lilly, IN, USA) for one week. BrdU incorporation into the DNA was determined by measuring the absorbance at 450 on an ELISA plate reader.

### Analysis of cell cycle distribution by flow cytometry

Cells were incubated 24 h with either 10 nM Paclitaxel (Bristol-Myers Squibb, New York, USA) or 1 nM vinorelbine (Pierre Fabre medicaments, Boulogne, France). Treated and untreated cells were then collected and incubated 1 hour at 4°C with propidium iodide (0.05 mg/ml) solution containing Nonidet-P40 (0.05%). Cells were analyzed using a FACS Calibur flow cytomoter (BD Biosciences Europe, Erembodegem, Belgium) and cell cycle distribution was determined using Modfit LT 2.0™ software (Veritysoftware Inc, Topsham, USA). For the siRNA's transfected clones, the cell cycle was studied 48 h after the first transfection.

### Long time-lapse microscopy and analysis of mitosis

Cells (3 × 10^5^) were seeded in a 35 mm cell culture dish, placed in culture medium maintained at 37°C in a 5% CO_2 _atmosphere and observed using an inverted time lapse microscope (Olympus IX50) at the Centre Commun de Quantimétrie (Université Claude Bernard Lyon, France). Images were acquired every 2 minutes for 24 hours using a numerical CFW-1308M 1360X1024 camera (Scion, Frederick, USA) driven by ImageJ software (NIH, Bethesda, USA). 30 complete mitoses were analysed for each of the MP6.1 and MC+1 clones using ImageJ software (NIH, Bethesda, USA).

### Immunofluorescence

Cells (MP6.1 and MC+1) exposed or not to 10 nM of paclitaxel for 24 hours were fixed by 4% paraformaldehyde during 15 minutes at room temperature and permeabilized using a PBS-Triton X-100 0.1% solution. Non specific sites were blocked using a solution containing 0.1% bovine serum albumin and 1% fetal calf serum. Cells were incubated with either a 1:100 of an antibody against β-tubulin (clone 2.1, Sigma Aldrich) or a 1:30 dilution of a monoclonal antibody against TBCC (Abnova, Taiwan) followed by a secondary FITC-antibody (Dako, Denmark). DNA staining was performed using diaminido-phenyl-indol (DAPI) (Roche, Manheim, Germany).

Images were obtained using a laser scanning confocal TCS Sp2 DMRXA microscope x63 objective (Leica Microsystems; Wetzlar, Germany) at the Centre Commun de Quantimétrie (Université Claude Bernard Lyon, France).

### Separation and quantification of soluble unfolded tubulins, polymerizable αβ-tubulin heterodimers and microtubules

Cells (20 × 10^6^) were harvested and lysed in 200 μl of buffer (100 mM Pipes, pH 6.7, 1 mM EGTA, and 1 mM MgSO_4_) by two freeze-thaw cycle. Lysed cells were centrifuged at 12,000 × g for 15 minutes at 4°C. The supernatant was then ultracentrifuged (100,000 × g for 1 h at 20°C) and separated into a supernatant containing "soluble tubulins" and a pellet containing "microtubules". The microtubule fraction was resuspended in 100 μl of lysis buffer and 100 μl of the supernatant were incubated with 1 mM of GTP at 35°C for 30 minutes to allow tubulin polymerization then ultracentrifuged at 50,000 × g for 45 minutes at 35°C. The resulting pellet contained the "polymerizable tubulin" (PT) heterodimers and the supernatant contained "nonpolymerizable tubulin" (NPT) heterodimers, included tubulin peptides complexed with tubulin binding cofactors. The different fractions of tubulins were run on silver stained gels following manufacturer's recommendations (Amersham Biosciences AB, Sweden). After coloration, the single band observed at 55 kDa for the polymerizable tubulin heterodimers confirmed the success of purification (data not shown). The experiment was performed in triplicate using the two cell lines MP6.1 and MC+1. Densitometric quantification of western blots was performed with ImageJ software (NIH, USA).

### Time lapse fluorescent microscopy and analysis of microtubule dynamics

Cells (3 × 10^5^) were seeded in 6-well plate with circular glasses of 24 mm in their bottom and transfected with the pAcGFP1-tubulin vector (Clontech) using lipofectin (Invitrogen) following the manufacturer's instructions. The glasses containing the cells were placed in culture medium maintained at 37°C in a 5% CO_2 _atmosphere in a time lapse inverted microscope (Olympus IX50) at the Centre Commun de Quantimétrie (Lyon, Université Claude Bernard, France). Cells were imaged with a numerical CFW-1308M 1360X1024 camera (Scion, Frederick, USA) driven by imageJ software (NIH, Bethesda, USA) using a 40× oil immersion lens (Zeiss, Göttingen, Germany). 30 pictures of the cell's microtubules were taken at 4 seconds intervals. The positions of the plus-ends of individual MT in peripheral lamellar regions of cells were tracked over time using ImageJ^® ^software and graphed using Microsoft^® ^Excel spreadsheet as position versus time to generate a 'life-history plot' for each MT. Growth and shortening rates and durations were derived by regression analysis. A difference of >0.5 μm between any two consecutive points was considered as a growth or shortening event. Transitions into depolymerisation or shortening are termed catastrophes, and transitions from shortening to growth or pause are called rescue. The catastrophe and rescue frequencies per unit time were calculated by dividing respectively the number of transitions from growth and pause to shortening and the number of transitions form shortening and pause to growth by the sum of the time in growth and pause. Dynamicity represents total tubulin exchange at the MT end and was calculated by dividing the sum of total length grown and shortened by the MT life span. The experiment was performed twice on 50 microtubules of the two clones MP6.1 and MC+1.

### *In vivo *growth analysis

Female CB17/SCID mice purchased from Charles River Laboratories (Arbresle, France) were bred under pathogen-free conditions at the animal facility of our institute. Animals were treated in accordance with the European Union guidelines and French laws for the laboratory animal care and use. The animals were kept in conventional housing. Access to food and water was not restricted. All mice used were 5 to 6 weeks old at the time of cells injections. This study was approved by the local animal ethical committee. Mice were divided into six groups of six mice each which corresponds to the injections of MP6.1, MP6.2, MP6.3, MC+1, MC+2 and MC+3 cells. 3 × 10^6 ^cells were injected subcutaneously in mice with 50% matrigel (BD Biosciences, Belgium). The six mice were divided into two groups of treated and untreated mice. In the treated groups, paclitaxel was injected intraperitoneally in a dose of 10 mg/kg on the same day and a week after. Mice were weighed and the tumor size was measured twice per week with an electronic caliper. The volume was then computed by considering the tumor as a sphere with the formula 4/3 (3.14 × r^3^), r as the mean radius. Animals were euthanized either when one of the diameters of the tumor exceeded 17 mm, or if any potential suffering of the animal was observed or if weight loss exceeded 10%.

## Results

### TBCC protein expression in stable clones

**Figure 1:**
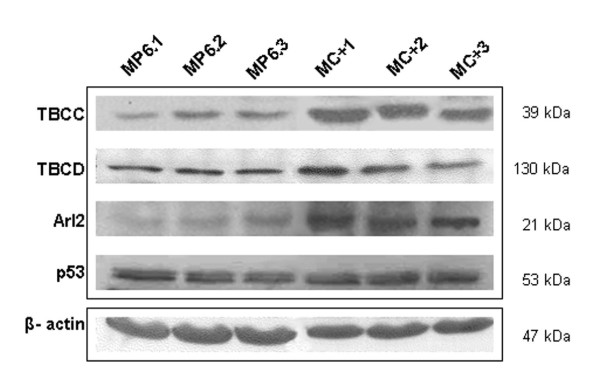
**Effect of tubulin binding cofactor C (TBCC) on the expression level of related proteins**. Western blot analysis of total cell extracts of the clones showing the effect of overexpression of TBCC on the expression level of TBCC, TBCD, Arl2 and p53. β-actin was used as a reference protein. MC+1, MC+2 and MC+3 represent MCF7 cells stably transfected with pcDNA6/C+. MP6.1, MP6.2 and MP6.3 represent MCF7 cells stably transfected with empty pcDNA6.

Six stable clones of MCF7 cells overexpressing the protein TBCC (designated MC+) were established and characterized compared to clones of control cells transfected with the empty vector (designated as MP6). Among these, three clones with high levels of TBCC, with respect to three clones of control cells, were named MC+1, MC+2 and MC+3 in decreasing order of expression and further explored (Figure 1). Three clones obtained from MCF7 transfected with the empty vector were designated MP6.1, MP6.2 and MP6.3 and used as controls.

### Impact of TBCC overexpression on the expression level of related proteins

We have investigated the effect of TBCC overexpression on the protein content of TBCD and ADP-ribosylation factor-like 2 (Arl2) which are two essential partners of TBCC in the folding pathway of α/β-tubulin dimers. TBCD was increased in the MC+1 clone but not in the two other clones MC+2 and MC+3. Conversely Arl2 was increased in the three clones of MC+ with respect to the three control clones of MP6 (Figure 1). Finally, we studied the expression level of the tumor suppressor protein p53 and found that it was slightly increased in the three clones of MC+ cells when compared to MP6 cells (Figure 1).

### Influence of TBCC protein content on proliferation rate

**Figure 2:**
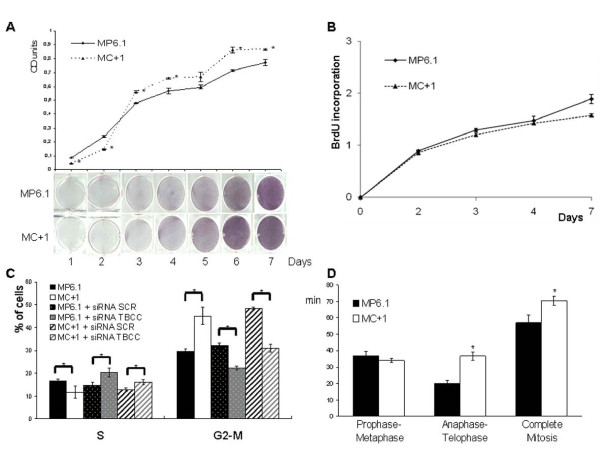
**Effect of TBCC on proliferation rate, cell cycle distribution and mitotic duration of MC+1 and MP6.1 cells**. (A) Optical density of absorbances measured at 540 and 690 nm of solubilized formazan crystals in MC+1 and MP6.1 formed after addition of MTT. The figure below the graph represents the MC+1 and MP6.1-containing wells colored with formazan crystals after addition of methylthiazoletetrazolium (MTT) every day for a total duration of one week. Results presented are the average values of three experiments. *: Values differ significantly from MP6.1 at ≥ 95% confidence level by Student's *t*-test. Bars represent standard deviation. (B) Proliferative capacity (BrdU labelling) of MC+1 and MP6.1 cells for one week. Results presented are the average values of three experiments. Bars represent standard deviation. (C) Cell cycle distribution of MC+1 and MP6.1 cells after incubation with propidium iodide. Percentage of cells in the S- and G2-M phase of the cell cycle in MC+1, MP6.1, MC+1 + siRNA SCR, MC+1 + siRNA TBCC, MP6.1 + siRNA SCR and MP6.1 + siRNA TBCC. The cells represented as +siRNA SCR and +siRNA TBCC are cells transiently double transfected with siRNA scrambled and siRNA targeting TBCC, respectively. Experiment was done 48 hours after first transfection. Results presented are the average values of three experiments. *: Values differ significantly at ≥ 95% confidence level by Student's *t*-test. Bars represent standard deviation. (D) Durations of prophase-metaphase, anaphase-telophase and complete mitosis in MC+1 and MP6.1 cells in minutes after 24 hours of time-lapse microscopic analysis. *: Values differ significantly from MP6.1 at ≥ 95% confidence level by Student's *t*-test. Bars represent standard deviation.

When we tested by MTT assay the proliferation rate of the MC+1 and MP6.1 cells *in vitro*, we found that the MC+1 cells had a slightly higher proliferation rate *in vitro *than the control cells at 120 and 144 hours but not at earlier timepoints. This result was visually confirmed by comparing the number of dividing cells stained in blue due to the formation of formazan crystals by their active mitochondria. These observations were confirmed by quantification of the absorbance optical density and the curve plotted (Figure 2A) shows the significant differences between these two cell lines. These results were confirmed on the MC+2, MC+3, MP6.2 and MP6.3 clones. When we tested the proliferation rate of MC+1 and MP6.1 cells using the BrdU incorporation assay, we found no significant difference in the proliferation rate between the two clones. This was confirmed on the other clones MC+2, MC+3, MP6.2 and MP6.3 (Figure 2B).

### Influence of TBCC protein content on cell cycle distribution and mitotic duration

To explore if the difference in proliferation rates is correlated to a difference in the cell cycle distribution, we investigated the percentage of cells in S and G2-M phases by propidium iodide and flow cytometry in MC+1 and MP6.1 cells (Figure 2C). We observed that MC+1 cells had a lower percentage of cells in S-phase 11.7 ± 2.6 as compared to MP6.1 cells 16.7 ± 0.78, a significant decrease of 30%, p < 0.05 (Figure 2C). This difference was also accompanied by a higher percentage of cells in G2-M phase 45 ± 3.9 as compared to the MP6.1 30 ± 1.27, a significant increase of 50%, p < 0.05 (Figure 2C). This experiment was performed on the MP6.2, MP6.3, MC+2 and MC+3 clones and the above results were confirmed.

In order to correlate these phenotypic behaviours to the increased content of TBCC protein, we inhibited the protein expression of TBCC by transient transfection using siRNA targetting TBCC in MP6.1 and MC+1 cells. The siRNA targetting TBCC we designed was validated by western blot on MCF7 cells. This sequence inhibited TBCC (-48%, p < 0.01) compared to the siRNA scrambled (data not shown) and was used to inhibit TBCC in MC+1 and MP6.1. In both the MC+1 and MP6.1 cells, the inhibition of TBCC protein caused a significant increase in percentage of cells in S-phase and a significant decrease in percentage of cells in G2-M phase (Figure 2C).

To explain the increased G2-M percentage observed in MC+1 cells, we investigated the durations of prophase-metaphase, anaphase-telophase and complete mitosis in comparison to controls. We found that MC+1 cells took more time to complete mitosis (71 minutes as compared to 57 minutes in MP6.1 cells). This longer time was due mainly to longer anaphase-telophase duration as 37 minutes compared to 20 minutes in MP6.1 and not to a difference in the prophase-metaphase (Figure 2D). The cells overexpressing TBCC proceed slowly through the mitosis which can be an explanation for the high percentage of cells in G2-M phase of cell cycle.

### *In vivo *progression of tumors

**Figure 3:**
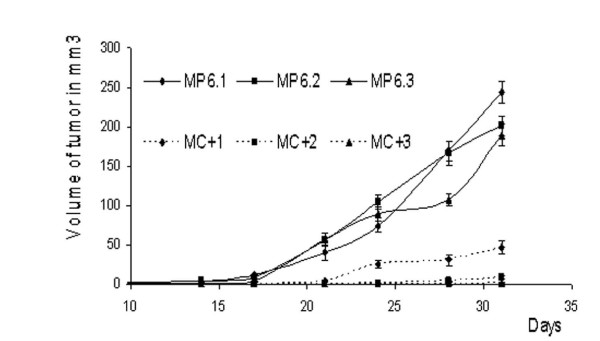
**In vivo tumor progression**. Progression of tumors growth measured at 2-3 days interval for one month after subcutaneous injections of each of MC+1, MC+2, MC+3, MP6.1, MP6.2 and MP6.3 cells. Results presented are average values of three mice. Bars represent standard deviation.

In order to study the impact of TBCC on tumorigenesis of breast cancer cells, we injected the six clones MP6.1, MP6.2, MP6.3, MC+1, MC+2 and MC+3 into SCID mice and monitored their *in vivo *progression and tumor formation. The control clones were able to form tumors of 200 to 250 mm^3^one month later. However, the MC+ clones presented lower capacities of growth *in vivo *with the tumors of volumes ranging form 1 to 50 mm^3 ^(Figure 3).

### Localisation of TBCC in the cytoplasm

**Figure 4:**
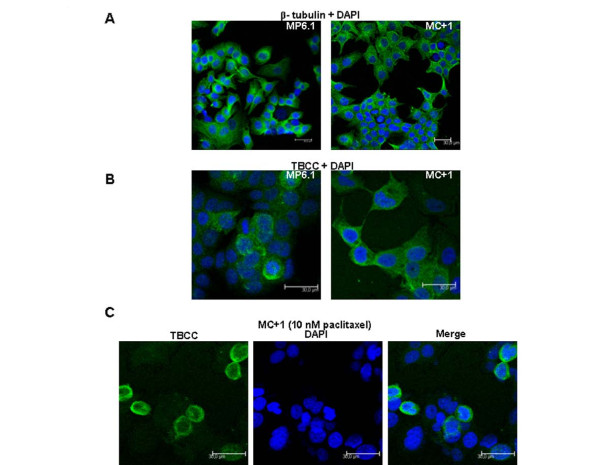
**Localization of TBCC in MC+1 and MP6.1 cells**. (A) Representative images of MC+1 and MP6.1 cells after DNA (DAPI, blue) and β-tubulin (FITC, green) staining (B) Representative images of MC+1 and MP6.1 cells after DNA (DAPI, blue) and TBCC (FITC, green) staining (C) Representative images of DNA (DAPI, blue) and TBCC (FITC, green) staining in MC+1 cells exposed 24 hours to 10 nM of paclitaxel.

The impact of TBCC overexpression on the cytoplasmic distribution of TBCC was studied by immunofluorescence on MP6.1 and MC+1 cells. In a first step we observed that the distribution of β-tubulins in the cytoplasm was not affected by the overexpression of TBCC. This was done by immunofluorescence using an antibody against β-tubulin and the images show similar cytoplasm staining between MP6.1 and MC+1 cells (Figure 4A). TBCC was similarly distributed in both normally and highly-expressing cells. The staining was found to be cytoplasmic however it was not observed in all cells of each cell line (Figure 4B). We found more stained cells in MC+1 cells than in control cells. After overlaying fluorescent images of TBCC with DAPI images we observed that the cells that were strongly stained for TBCC were cells in mitosis. This result suggested that TBCC was more highly expressed in cells undergoing mitosis. This was confirmed (Figure 4C) in MC+1 cells exposed to paclitaxel 10 nM for 24 hours. Paclitaxel is known to stabilize microtubules and causes cell arrest in metaphase. We observed by immunofluorescence microscopy that the cells blocked in mitosis were strongly stained with anti-TBCC antibody.

### Effect of TBCC content on different tubulins and on subcellular tubulin fractions

**Figure 5:**
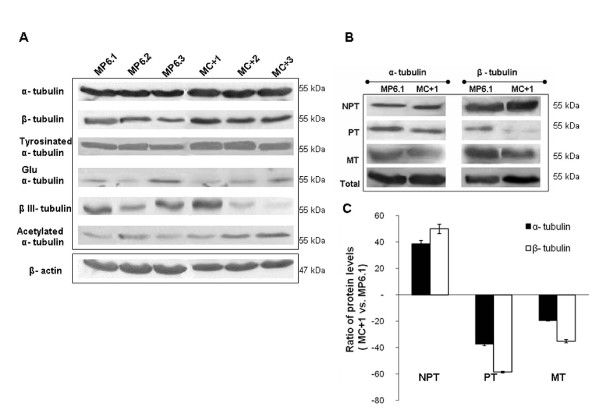
**Effect of TBCC on different tubulins content and on subcellular tubulin fractions**. (A) Western blot analysis of total cell extracts of the MC+1, MC+2, MC+3, MP6.1, MP6.2 and MP6.3 clones showing the effect of overexpression of TBCC on the expression level of α-tubulin, β-tubulin, tyrosinated α-tubulin, detyrosinated (glu) α-tubulin, β III-tubulin and acetylated α-tubulin. β-actin was used as a reference protein. (B) Representative blots corresponding to the expression levels of α-tubulin and β-tubulin in nonpolymerizable tubulin heterodimers (NPT), polymerizable tubulin (PT) heterodimers, microtubule heterodimers (MT) and total pool of tubulins in MC+1 and MP6.1 cells. These fractions were obtained after series of ultracentrifugations. (C) Protein ratios (MC+1 vs. MP6.1) of both α-tubulins and β-tubulins levels in NPT, PT and MT fractions. Results presented are the average values of three experiments. Bars represent standard deviation.

We studied the impact that TBCC overexpression might have on the total α and β-tubulins as well as on different posttranslational modifications of α-tubulin such as acetylated, tyrosinated and detyrosinated (Glu) α-tubulins and finally on the βIII-tubulin isotype. We observed no significant difference in the expression of total α-tubulin and tyrosinated α-tubulin between the clones overexpressing TBCC and the controls (Figure 5A). Regarding Glu α-tubulin and the βIII-tubulin, we observed heterogeneity of expression among both the clones of MP6 and MC+ which made impossible to conclude of a simple profile related to TBCC status (Figure 5A). However, concerning β-tubulin and acetylated α-tubulin we observed an increase in expression in the MC+ clones with respect to the MP6 clones.

In addition, we investigated the different pools of soluble polymerizable αβ-heterodimers (PT) and soluble non-polymerizable tubulins (NPT) as well as the microtubule tubulins (MT) in MC+1 and MP6.1 cells. The different pools of tubulins were obtained after fractionation of MP6.1 and MC+1 lysates by series of ultracentrifugations. We have observed that the modification of the TBCC level in MCF7 cells did not alter the total pool of α-tubulin protein although it increased the total pool of β-tubulin (Figure 5B). The overexpression of TBCC protein strongly increased the NPT fraction and strongly decreased the PT fraction, while it had only a minor effect on the tubulin content of microtubules which was slightly decreased. The profile of expression of the NPT and PT fractions was similar for both the α-tubulins and the β-tubulins but was less marked in the case of α-tubulins (Figure 5B). Quantification of expression levels of α and β-tubulins in the different fractions confirmed the results observed in the immunoblots (Figure 5C).

### Overexpression of TBCC decreases microtubule dynamics

The effects of overexpression of TBCC on microtubule dynamic instability were determined in MC+1 cells 48 h after transient transfection with pAcGFP1-α tubulin. The microtubules in MC+1 cells grew and shortened more slowly and for shorter lengths than those in MP6.1 cells and presented significantly less dynamicity. The parameters for dynamic microtubules in MP6.1 and MC+1 cells were determined (Table 1). The mean growth rate was significantly decreased from 16.41 ± 1.99 μm/min in MP6.1 cells to 10.54 ± 0.37 μm/min in MC+1 cells, corresponding to a decrease of 36%, p < 0.05. The mean shortening rate and the mean lengths of individual growth and shortening were slightly decreased. The mean shortening duration was slightly increased compared to the mean growth duration and mean pause duration that were significantly increased from 0.15 ± 0.02 min and 0.25 ± 0.01 min in control cells to 0.22 ± 0.01 min and 0.37 ± 0.02 min in MC+1 cells, an increase of 47%, p < 0.05 and 48%, p < 0.05, respectively. The MC+1 cells presented a slower growth movement than the control cells which is the cause of a slow growth rate and a lower dynamicity. The frequencies of catastrophe and rescue are important parameters in studying the dynamicity of microtubules. The catastrophe frequency is the frequency with which the microtubules switch from either pause or growth to shortening. The rescue frequency is the frequency with which the microtubules switch from shortening to either growth or pause. As seen above, the MC+1 cells presented a shortening rate similar to that of the control cells but with a decreased growth rate. This suggests that the microtubules shortened in MC+1 cells are either taking longer time to grow (high growth duration) or staying in a pause phase (high pause duration). The time-based catastrophe frequency was slightly increased in MC+1 cells unlike the rescue frequency that was increased significantly by 54%, p < 0.05 as compared to control cells. This can be explained by the fact that the microtubules of MC+1 cells have tiny movements of growth and shortening for short distances (less than 0.5 μm) not counted in the growing and shortening events and in the overall dynamicity of the microtubule. The dynamicity was significantly decreased from 8.80 ± 1.02 μm/min in MP6.1 to 6.09 ± 1.06 μm/min in MC+1 cells. This decrease of 31%, p < 0.05 is representative of less overall distances travelled by the microtubule either in a growing or in a shortening event for 2 minutes.

**Table 1:** Parameters of microtubule dynamics in MC+1 and MP6.1 cells

Parameters		MP6.1	MC+1	Change
Mean rate (μm/min ± SE)	Growth	16.41 ± 1.99	10.54 ± 0.37 *	-36%
	Shortening	15.25 ± 2.14	14.35 ± 1.53	
Mean length (μm ± SE)	Growth	2.38 ± 0.49	2.14 ± 0.51	
	Shortening	2.40 ± 0.39	2.29 ± 0.10	
Mean duration (min ± SE)	Growth	0.15 ± 0.02	0.22 ± 0.01 *	47%
	Shortening	0.16 ± 0.01	0.17 ± 0.01	
	Pause	0.25 ± 0.01	0.37 ± 0.02 *	48%
Mean frequency (min^-1^± SE)	Rescue	9.20 ± 0.53	14.20 ± 0.24 *	54%
	Catastrophe	4.64 ± 0.70	5.38 ± 0.56	
Dynamicity (μm/min ± SE)		8.80 ± 1.02	6.09 ± 1.06 *	-31%

*: Values differ significantly from MP6.1 at ≥ 95% confidence level by Student's *t*-test. Values are represented as mean ± SD

### *In vitro *response to Gemcitabine and antimicrotubule compounds

**Figure 6:**
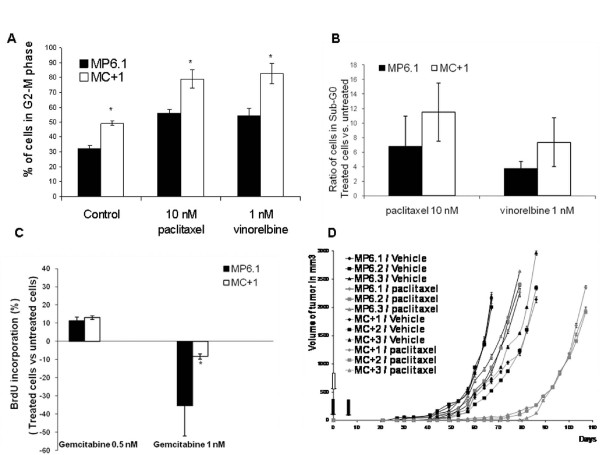
**The influence of TBCC protein content on the response to treatment in vitro and in vivo**. (A) The Percentage of MC+1 and MP6.1 cells in the G2-M phase of the cell cycle after 24 hours of treatment with 10 nM of paclitaxel and 1 nM of vinorelbine. Results presented are the average values of three experiments. Bars represent standard deviation.*: Values differ significantly from MP6.1 at ≥ 95% confidence level by Student's *t*-test. (B) The ratio of treated vs. untreated MC+1 and MP6.1 cells in the Sub-G0 phase of the cell cycle after 24 hours of treatment with 10 nM of paclitaxel and 1 nM of vinorelbine. The cell cycle was studied after incubation with propidium iodide. Results presented are the average values of three experiments. Bars represent standard deviation. (C) Proliferative response by BrdU labelling (treated versus untreated cells) after one week exposure to 0.5 and 1 nM of gemcitabine in MC+1 and MP6.1 cells. Results presented are the average values of three experiments.*: Values differ significantly from MP6.1 at ≥ 95% confidence level by Student's *t*-test. Bars represent standard deviation. (D) Progression of tumors growth and response to paclitaxel measured at 2-3 days interval after subcutaneous injections of each of MC+1, MC+2, MC+3, MP6.1, MP6.2 and MP6.3 cells at day 0 (empty arrow). At day 0 and day 7, paclitaxel was injected intraperitoneally in a dose of 10 mg/kg (black arrow). Results presented are the mean values of the three mice, untreated or treated. Bars represent standard deviation.

Since TBCC is a major protein involved in the proper folding pathway of tubulins into microtubules, we investigated the response of the TBCC overexpressing breast cancer cells to antimicrotubule agents. These agents block the cell cycle in G2-M phase. We incubated MC+1 and MP6.1 cells 24 hours with non-toxic doses of 10 nM paclitaxel or 1 nM vinorelbine, and observed that the cells overexpressing TBCC were more sensitive to the G2-M blockage caused by these treatments with respect to the MP6.1 cells. MC+1 cells presented 79 and 83% of cells in G2-M after paclitaxel and vinorelbine exposure, respectively. The control cells were also blocked but to a maximum percentage of 55% (Figure 6A). The percentage of MC+1 and MP6.1 cells in the Sub-G0 phase of the cell cycle after 24 hours of treatment with 10 nM of paclitaxel and 1 nM of vinorelbine increased. However this increase was not statistically significant and was slightly higher in MC+1 cells than in MP6.1 cells (Figure 6B). In addition to antimicrotubule agents, we investigated the response of the cells to gemcitabine, an S-phase specific antimetabolite and we found that MC+1 cells were less sensitive to gemcitabine than the MP6.1 cells, significantly at the dose of 1 nM (Figure 6C). These experiments were performed on the MP6.2, MP6.3, MC+2 and MC+3 clones and the above results were confirmed.

### *In vivo *sensitivity to treatment

We injected the six clones MP6.1, MP6.2, MP6.3, MC+1, MC+2 and MC+3 into SCID mice and monitored their *in vivo *progression and tumor formation. The control clones were able to form tumors unlike the MC+1, MC+2 and MC+3 clones that presented lower capacities of growth *in vivo*. When treated with paclitaxel, mice injected with MC+ clones revealed higher sensitivity to the treatment compared to mice injected with MP6 clones (Figure 6D).

## Discussion

Tubulin binding cofactor C is a crucial protein for the proper folding of α- and β-tubulins to form heterodimers able to polymerize into microtubules. In this study, the major aim was to investigate the impact of TBCC overexpression on the proliferation, cell cycle distribution and tumorigenesis of MCF7 cells as well as on the microtubule contents and dynamics. Since the antimicrotubule agents are common treatments for breast cancer, we examined the response of our models to these treatments both *in vivo *and *in vitro*. In addition to this, expression levels of α and β tubulins were found to be involved in predicting the response to treatments in many cancers [22,23]. Some posttranslational modifications of α tubulins like detyrosination are of high occurrence in breast cancer [24]. This differential expression level of tubulins in many cancers has made of them targets for treatments.

The overexpression of TBCC in MCF7 cells profoundly altered the distribution of tubulin monomers amongst cellular fractions and diminished the content and dynamicity of their microtubules but did not prevent the cells from completing mitosis and proliferating correctly. We must insist however on the fairly low differences in TBCC content observed between transfected cells and controls, suggesting that higher levels of expression may be incompatible with cell survival. Overexpression of TBCC had a major impact on tubulin fractions, with a large increase in the nonpolymerizable fraction and a consequent decrease in the soluble tubulin dimers fraction. The nonpolymerizable tubulin fraction consists of the pool of tubulins in the α-tubulin/TBCE/TBCC/TBCD/β-tubulin complex, the pool of α-tubulins bound either to TBCB or TBCE or TBCE/TBCC/TBCD/β-tubulin, and the pool of β-tubulins bound either to TBCA or TBCD or α-tubulin/TBCE/TBCC/TBCD. The reduced availability of α/β tubulins to form polymerizable heterodimers may be due to the fact that a large amount of the monomers is included in TBCC-containing complexes. Of note and contrary to a commonly accepted tubulin dogma, MC+ cells appear to have disequilibrium between the contents of total α-tubulin and β-tubulin. It is classically considered that such disequilibrium would be lethal for mammalian cells. However in this model the increased content can be attributed to a specific enrichment in the non-functional fraction of β-tubulin.

During mitosis, especially at the level of anaphase chromosome movement, microtubules disassemble by depolymerization and release free heterodimers. The heterodimers released can be directly recruited by the excess of TBCC in the cytoplasm. Indeed, TBCC appears to be highly present in the cytoplasm during mitosis as observed in cells blocked in mitosis by paclitaxel. We hypothesize that equilibrium exists between the soluble amount of nonpolymerizable and polymerizable tubulins in MC+ cells and the cells' requirement for microtubules. We also observed a strong impact of TBCC content on microtubule dynamics. At the onset of mitosis the interphase microtubule network disassembles while there is simultaneously a decrease in total microtubule polymer mass and an increase in microtubule dynamics [25]. In some cells, the increase in dynamics is due to an increase in catastrophe frequency and a reduction in the rescue frequency rather than changes in growth and shortening rates [26,27]. In our study, we found that the microtubules in MC+1 cells grew and shortened more slowly and for shorter periods of time than those in MP6.1 cells. The dynamicity was significantly decreased in MC+1 cells in comparison to MP6.1 cells. These observations are coherent with the tubulin fraction alterations observed. The relative lack of available polymerizable tubulin dimers could explain the reduced growing and overall dynamicity.

The highly dynamic microtubules in the spindle are required for all stages of mitosis [2]. During prometaphase, the dynamicity of microtubules is very important in order to probe the cytoplasm and attach to chromosomes at their kinetochores [28]. Any single chromosome unable to attach to the spindle is enough to prevent a cell from transitioning to anaphase and therefore be blocked at or before metaphase-anaphase transition and undergo apoptosis later on [29,30]. In our study, the decreased dynamicity in MC+ cells did not affect the prophase and metaphase progression and only affected the anaphase-telophase transition. We observed a significant increase in the duration of anaphase-telophase which is the cause of a slower mitosis in the MC+ cells. This means that the less dynamic microtubules affected the mitosis without causing blockage or cell death. The distribution of MC+ cells in the cell cycle was different from that of the control cells in that they presented higher percentage of cells in the G2-M phase and lower percentage in the S-phase. By inhibiting TBCC protein through transient transfection of siRNA targeting TBCC, we obtained an increase in the percentage of cells in S-phase and a decrease in the percentage of cells in G2-M, thereby confirming the involvement of TBCC in the cell cycle alteration observed.

Previous publications have suggested that the dynamicity of microtubules depends on microtubule composition and is correlated with post-translational modifications of α-tubulins. Detyrosinated microtubules (Glu microtubules) present enhanced stability against end-mediated depolymerisation however the detyrosination alone is not sufficient to confer this enhanced stability [31]. Tubulin detyrosination occurs frequently in breast cancer and is linked to tumor aggressivity [24]. Reduced abundance of α and α-acetylated tubulin is associated with enhanced apoptosis in leukemia cells [32]. Acetylated α-tubulin is present in microtubules that under depolymerising condition are more stable than the majority of cytoplasmic microtubules [31,33]. The existence of a direct effect of acetylation on microtubule stability and dynamics remains controversial [34]. In our study, we investigated the expression levels of detyrosinated (Glu) α-tubulin, tyrosinated α-tubulin and acetylated α-tubulin. Reproducible results were obtained with the tyrosinated tubulin which was not modified and for the acetylated tubulin that was increased in the MC+ cells with respect to MP6 cells. The results of increased expression level of acetylated α-tubulin in MC+ cells can be explained as an attempt of these cells to protect their microtubules. The microtubules in MC+ cells have diminished dynamicity and their reduced growth rate is not enough to compensate for their continuous shortening events. As a means to prevent excessive depolymerisation, the microtubules of MC+ might have incorporated acetylated α tubulin in order to acquire more stability against depolymerisation. It has been previously reported that high expression of class III beta tubulin by tumor cells is associated with resistance to taxane chemotherapy in non-small cell lung cancer [35]. Here we can't do any correlation, since the expression profile of the class III beta tubulin in our models is not determined.

The MCF7 cells (human mammary adenocarcinoma) emerge from an invasive ductal carcinoma type of cancer [36,37]. The *in vivo *invasiveness and metastasis processing of MCF7 cells depend on many factors that influence the cell such as steroid hormones, growth factors, oncogenes and tumor suppressor genes [38]. In our study, MC+ cells presented a limited tumor growth *in vivo *compared to MP6 cells. Since the proliferative activity of MC+ cells *in vitro *was not reduced in comparison to control cells and their volume was not altered (data not shown), we hypothesize that this reduced *in vivo *growth may be at least partially explained by the potential loss of aggressive and invasive capacities of MC+ cells rather than by decreased proliferation rate [39]. The study of expression levels of TBCC, α tubulin and β tubulin in the tumors extracted from the mice revealed that the MC+ cells maintained the same alterations *in vivo *as those reported *in vitro *(data not shown). In the intent to understand the potential involvement of TBCC in the invasiveness phenotype, we have studied by quantitative RT-PCR the levels of expression of the *TBCC *gene in thirteen different human breast cancer cell lines for which *in vitro *invasiveness properties have been reported [40]. We found that the four cell lines that expressed *TBCC *the highest (MCF7, MDA-MB361, MDA-MB453 and UACC812) were the ones with low *in vitro *invasiveness capacity and the other nine cell lines that were highly invasive had low *TBCC *expression (Additional file 1). While this observation does not allow us to conclude a direct role of TBCC it suggests a possible involvement of TBCC in tumor aggressivity whether through microtubules or through other unidentified pathways such as interaction with the Arl2 protein. It is important to note that in the cells overexpressing TBCC we noticed that the expression levels of Arl2 and tumor suppressor p53 were slightly increased. ADP ribosylation factor like 2 (Arl2) protein is a GTPase that belongs to ADP ribosylation factor (ARF) family [41,42] and plays a role in microtubule dynamics [21,42]. Arl2 is known to directly bind to TBCD and can inhibit TBCD from dissociating the α/β tubulin heterodimers [17]. MCF7 cells overexpressing Arl2 were found to have low i*n vivo *growth capacity [43]. However *in vitro*, the behaviour of MC+ cells in terms of cell cycle, microtubule dynamics, response to antimicrotubule agents largely differ from that of cells overexpressing Arl2 [21]. Therefore one possible hypothesis could be that *in vivo *a mechanism involving TBCC and p53 or an interaction between Arl2 and TBCC is involved in the loss of aggressivity of MC+ cells.

Based on the success and efficiency of microtubule-targeted drugs in the treatments of cancer in general and breast cancer in specific, microtubules remain the best cancer target identified to date [44]. Even though paclitaxel and vinorelbine have different mechanisms of action with respect to microtubule, their cellular effects at low but clinically relevant concentrations are reduced microtubule dynamics inducing mitotic arrest. Paclitaxel, from the taxanes family binds to β-tubulin, causes lateral polymerization and suppresses microtubule dynamics [45]. The cellular effect of paclitaxel at low concentrations (<10 nM) include suppression of microtubule dynamics without affecting microtubule content and mitotic arrest then apoptosis [46]. Vinorelbine, from the vinca alkaloids binds with high affinity to the plus end of the microtubule and with low affinity to the sides of the microtubule and leads to depolymerization [46,47]. However at low concentrations, vinca alkaloids block mitosis with little or no depolymerisation of spindle microtubules [48]. Therefore, compounds that depolymerize microtubules can also stabilize microtubule dynamics at relatively low concentrations [49]. In our study, we have observed an increased sensitivity of the MC+ cells *in vitro *toward the mitosis blockage compared to MP6 cells. The low non toxic concentrations of both paclitaxel (10 nM) and vinorelbine (1 nM) induced a stronger G2-M block in MC+ cells than in MP6 cells. We used subtoxic concentrations of treatments as shown by the absence of significant increase in percentage of sub-G0 cells. The results observed were homogenous in the three clones of each cell line. The enhanced sensitivity of MC+ cells to paclitaxel was confirmed *in vivo*. We explain the increased sensitivity of the MC+ for the antimicrotubule agents by two main hypotheses. One is the basal lower dynamicity of microtubules in these cells compared to MP6 cells. Second is the high percentage of G2-M cells in MC+ cells which means high percentage of target cells for antimicrotubule agents. These results suggest that TBCC content of breast tumor cells significantly influences their sensitivity to tubulin binding agents both *in vitro *and *in vivo*. When we tested the response of MC+1 and MP6.1 cells to gemcitabine, a nucleoside analog and S-phase specific antimetabolite, we showed that MC+1 cells were less sensitive to gemcitabine than the MP6.1 cells, significantly at 1 nM. This lower sensitivity to gemcitabine is due to the fact that MC+1 cells present a lower percentage of cells in the S-phase at the basal level. The differential responses to antimicrotubule agents and gemcitabine reveal that our cell models respond to these treatments based on their distribution in the cell cycle. Therefore we suggest that other anticancer treatments that target the cell cycle can have interesting effects on our cell models.

## Conclusion

Our results suggest that the overexpression of TBCC protein in MCF7 cells influences their tubulin pools and microtubule dynamics, with important consequences in terms of mitotic progression, tumor growth and sensitivity to antimicrotubule agents. Moderately increased TBCC content was associated with an increase in the nonpolymerizable tubulin pool and reduced microtubule dynamics. This was associated with prolonged anaphase-telophase and reduced growth *in vivo*. The higher percentage of cells in G2-M phase of the cell cycle made of the MC+ cells better targets for antimicrotubule agents. These results underline the importance of the microtubular network in the tumor cell aggressivity phenotype. While currently available compounds mostly target microtubule dynamics, another possibility could be to alter tubulin pools in tumor cells. Another perspective for these results would be to look for potential partners of TBCC. It would be interesting to investigate if TBCC interacts or binds with microtubule binding proteins and to try to synthesize a ligand that can stabilize this protein inside the cytoplasm so that it would be continuously active.

## Competing interests

Rouba Hage-Sleiman benefits from financial support from the Lebanese CNRS.

## Authors' contributions

RHS and CD conceived and designed the experiments. RHS and CD wrote the manuscript. RHS and SH performed the *in vivo *studies and analyzed the data. RHS, SH, ELM and JFL performed the *in vitro *studies. RHS analyzed the *in vitro *data. CD contributed with reagents/materials/analysis tools. All authors read and approved the final manuscript.

## Pre-publication history

The pre-publication history for this paper can be accessed here:

http://www.biomedcentral.com/1471-2407/10/135/prepub

## Supplementary Material

Additional file 1 **Correlation between *TBCC *expression level and *in vitro *invasive capacity of breast cancer cell lines**. Values of gene expression are calculated with respect to the *TBCC *expression level in HME cells (human mammary epithelial cells). The cDNA levels were normalized to the expression of the 18S ribosomal gene as previously described by Saussede-Aim et al. 2009. Saussede-Aim J, Matera EL, Herveau S, Rouault JP, Ferlini C, Dumontet C: **Vinorelbine Induces β3-Tubulin Gene Expression through an AP-1 Site**. *Anticancer research *2009, 29:3003-3009. Click here for file
