# Supplementary material for: Tubulin binding cofactor C (TBCC) suppresses tumor growth and enhances chemosensitivity in human breast cancer cells
Source: BMC Cancer. 2010 Apr 12;10:135. doi: 10.1186/1471-2407-10-135 (PMC2859754; doi:10.1186/1471-2407-10-135)
Supplement: Additional file 1 — Correlation between TBCC expression level and in vitro invasive capacity of breast cancer cell lines. Values of gene expression are calculated with respect to the TBCC expression level in HME cells (human mammary epithelial cells). The cDNA levels were normalized to the expression of the 18S ribosomal gene as previously described by Saussede-Aim et al. 2009. Saussede-Aim J, Matera EL, Herveau S, Rouault JP, Ferlini C, Dumontet C: Vinorelbine Induces β3-Tubulin Gene Expression through an AP-1 Site. Anticancer research 2009, 29:3003-3009. [file 1471-2407-10-135-S1.DOCX]

# Additional files

### Additional file 1

### Correlation between *TBCC* expression level and *in vitro* invasive capacity of breast cancer cell lines

| Cell line | *TBCC* Expression level | *In vitro* invasive capacity |
| --- | --- | --- |
| MCF7 | 4.10 | Low |
| UACC812 | 3.56 | Low |
| MDAMB361 | 3.49 | Low |
| MDAMB453 | 3.00 | Low |
| MDAMB436 | 2.79 | High |
| BT20 | 2.52 | High |
| HS578T | 2.31 | High |
| MDAMB157 | 1.71 | High |
| CAL51 | 1.55 | High |
| HBL100 | 1.02 | High |
| T47D | 1.00 | High |
| BT474 | 0.94 | High |
| MDAMB231 | 0.83 | High |

Values of gene expression are calculated with respect to the *TBCC* expression level in HME cells (human mammary epithelial cells).

The cDNA levels were normalized to the expression of the 18S ribosomal gene as previously described by Saussede-Aim et al. 2009.

Saussede-Aim J, Matera EL, Herveau S, Rouault JP, Ferlini C, Dumontet C: **Vinorelbine Induces β3-Tubulin Gene Expression through an AP-1 Site.** *Anticancer research* 2009, 29:3003-3009.
